# Neuropathological findings in Down syndrome, Alzheimer’s disease and control patients with and without SARS-COV-2: preliminary findings

**DOI:** 10.1007/s00401-024-02743-9

**Published:** 2024-05-27

**Authors:** Ann-Charlotte E. Granholm, Elisabet Englund, Anah Gilmore, Elizabeth Head, William H. Yong, Sylvia E. Perez, Samuel J. Guzman, Eric D. Hamlett, Elliott J. Mufson

**Affiliations:** 1 Department of Neurosurgery, University of Colorado Anschutz Medical Campus, Research Complex II, Aurora, CO USA; 2 Division of Pathology, Department of Clinical Sciences, Lund University, Lund, Sweden; 3 Department of Pathology and Laboratory Medicine, University of California Irvine, Irvine, CA USA; 4 Department of Neurology, University of California Irvine, Irvine, CA USA; 5 Department of Translational Neuroscience and Neurology, Barrow Neurological Institute, Phoenix, AZ USA; 6 Department of Pathology, University of Colorado Anschutz Medical Campus, Aurora, CO USA; 7 Department of Pathology and Laboratory Medicine, Medical University of South Carolina, Charleston, SC USA

**Keywords:** SARS-CoV-2, COVID-19, Corona viruses, Neurologic symptoms, Brain, Alzheimer’s disease, Glial cells, Down syndrome

## Abstract

The SARS-CoV-2 virus that led to COVID-19 is associated with significant and long-lasting neurologic symptoms in many patients, with an increased mortality risk for people with Alzheimer’s disease (AD) and/or Down syndrome (DS). However, few studies have evaluated the neuropathological and inflammatory sequelae in *postmortem* brain tissue obtained from AD and people with DS with severe SARS-CoV-2 infections. We examined tau, beta-amyloid (Aβ), inflammatory markers and SARS-CoV-2 nucleoprotein in DS, AD, and healthy non-demented controls with COVID-19 and compared with non-infected brain tissue from each disease group (total *n* = 24). A nested ANOVA was used to determine regional effects of the COVID-19 infection on arborization of astrocytes (Sholl analysis) and percent-stained area of Iba-1 and TMEM 119. SARS-CoV-2 antibodies labeled neurons and glial cells in the frontal cortex of all subjects with COVID-19, and in the hippocampus of two of the three DS COVID-19 cases. SARS-CoV-2-related alterations were observed in peri-vascular astrocytes and microglial cells in the gray matter of the frontal cortex, hippocampus, and para-hippocampal gyrus. Bright field microscopy revealed scattered intracellular and diffuse extracellular Aβ deposits in the hippocampus of controls with confirmed SARS-CoV-2 infections. Overall, the present preliminary findings suggest that SARS-CoV-2 infections induce abnormal inflammatory responses in Down syndrome.

## Background

SARS-CoV-2 is a coronavirus that gave rise to COVID-19, which spread rapidly throughout the world, resulting in the death of more than 6.9 million people during the pandemic of 2019–21 [16]. Neurologic side effects have been reported in numerous patients both with SARS-CoV-2 infections and its predecessors, SARS (severe acute respiratory syndrome) and MERS (Middle Eastern Respiratory Syndrome) [23, 46, 78]. Viruses enter the central nervous system (CNS) either through hematogenous transport, the blood–brain barrier (BBB), or retrograde axonal transport [81]. Since SARS, MERS, and SARS-Cov-2 are respiratory viruses that enter the body via aerosol droplets, access to the CNS could be via nerve terminals located in the upper respiratory tract, via olfactory mucosa, olfactory bulb and olfactory tract [7, 61]. However, whether this is the main pathway for CNS-invasion of SARS-CoV-2 is an issue that remains under-investigated. The primary cellular receptor for SARS-CoV-2 is the Angiotensin-converting enzyme 2 (ACE2) receptor [51]. A study reported that neurologic symptoms were exclusively found in patients with a moderate or high expression of ACE2 in peri-vascular cells, while similar symptoms were not detected in individuals without peri-vascular ACE2 [8].

Furthermore, viral particles of SARS-CoV-2 were discovered in *postmortem* cerebral spinal fluid (CSF) and brain gray matter [34, 41, 55, 61, 63, 75]. Several different clinical–pathologic aspects observed in patients with COVID-19 such as neurologic symptoms, frontotemporal hypoperfusion, frontal slowed EEG and frontal hypometabolism on ^18^F-FDG-PET imaging, suggesting that SARS-CoV-2 initially accumulates in the frontal lobes [30, 77]. An FDG-PET study showed that patients with cortical cognitive dysfunction associated with COVID-19 displayed hypometabolism in the frontal cortex, anterior cingulate, insula and caudate nucleus [48]. Furthermore, transcriptomics revealed SARS-CoV-2 viral activity in frontal cortex of COVID-19 patients [24]. Although the ability of the SARS-CoV-2 virus to enter the CNS may underlie the loss of smell and taste, headaches, fatigue, nausea, dizziness, and delirium reported by people that recovered from COVID-19 [27, 77], little is known about the pathogenesis of SARS-CoV-2 in the brain of people with neurodegenerative disorders.

Interestingly, the SARS-CoV-2 virus is associated with an exaggerated immune response, long-lasting neurologic symptoms, and an increased mortality risk for people with Alzheimer’s disease (AD, [35]) and/or Down syndrome (DS, [56]). Down syndrome is a developmental genetic condition associated with the most prevalent type of intellectual disability caused by complete or partial trisomy of human chromosome 21 (Chr 21) that affects more than 350,000 people in the USA alone [84]. Due to improved healthcare, individuals with DS have experienced a significant increase in average life span the last few decades but suffer from an increased incidence of early onset AD and associated amyloid [37], phospho-tau [38, 57, 72] and neurotransmitter cellular dysfunction [4, 11, 28, 32, 49, 54] compared to the general population [36, 79, 80]. Moreover, adults with DS have an estimated fourfold increased risk for COVID-19 hospitalization and a tenfold increased risk for COVID-19-related mortality [13]. This led the Centers for Disease Control (CDC) to place persons with DS on the priority list for COVID-19 vaccination, both in Europe and the USA. The increased vulnerability to COVID-19 in people with DS might be due to several factors: a) sensitivity to pulmonary infections due to anatomic and medical comorbidities [42], b) the presence of the gene encoding for transmembrane serine protease 2 (TMPRSS2), a protein involved in spike protein priming after cell entry [39] on Chr 21, c) increased inflammatory response in individuals with DS, possibly due to the observation that four of the six known genes encoding interferon receptors are located on Chr 21 [12, 18, 22, 73], and d) secretion of excessive amounts of exosomes [33] by which SARS-CoV-2 virus spreads [31]. Finally, persons with DS have a dysfunctional antibody response to viral respiratory infections [42], possibly increasing vulnerability to COVID-19. Despite these findings, there is a paucity of data on the neuropathological effect(s) and inflammatory reaction of the brain in people with DS after COVID-19. A single trisomy 21 case was listed in a study of the neuropathology of patients with COVID-19 from a German cohort showing widespread astrocytosis throughout the brain including the frontal cortex, but the study did not provide a detailed description of the DS case [59]. Others have reported microglial abnormalities in frontal cortex of patients with AD and COVID-19 [29], in the temporal and parietal cortex in elders with COVID-19 with dementia [66] and activated inflammatory signaling and oxidative overload in lateral cortex in patients with Covid [67]. It is generally thought that there is also an increased mortality rate for COVID-19 in patients with AD [83]. Moreover, SARS-CoV-2 infection induces AD tau pathology and increases AD plasma biomarkers [19, 21]. However, to date there are no studies that have compared glial pathology in the brain of individuals with COVID-19 and AD, DS, or DS-AD comorbidities. To our knowledge, the present study is the first in-depth analysis of glial pathology in *postmortem* brains of people with DS with or without pre-existing AD, patients with AD and aged controls that succumbed to the SARS-CoV-2 virus infection. Here, we provide evidence showing an abnormal microglial and astrocytic neuroinflammatory response as well as the presence of SARS-CoV-2 nucleoprotein antibodies in *postmortem* human frontal cortex and to a lesser degree in hippocampal tissue obtained from individuals with AD, DS, or controls with or without COVID-19.

## Materials and methods

### Demographic information

We examined brain tissue from three different clinical cohorts obtained from donations or clinical autopsies from 7 individuals with DS obtained from the Down Syndrome Biobank consortium (DSBC), a multisite brain bank consortium, a clinical autopsy cohort of 9 cases with or without COVID-19 from Lund University (Dr. Elisabet Englund), and 8 cases from the Medical University of South Carolina (MUSC) Brain bank in Charleston (Dr. Eric Hamlett). Table 1 details the demographic data for the 12 confirmed COVID-19 and 12 non-COVID-19 AD, DS and non-demented healthy control (CTRL) cases examined. The collection consisted of 10 females and 14 males with an average age of 58 years (range from 31 to 96 years) at the time of death**.** AD pathology varied across the different diagnostic groups: three AD non-COVID-19, four AD COVID-19 + , five non-COVID-19 and five COVID-19 + controls, one DS and two DS-AD without COVID-19 and three DS with COVID-19. Only one of the DS with COVID-19 reported dementia prior to the SARS-CoV-2 infection (case 24; DS COVID-19 recovered (DS COVID + r). Controls were carefully selected based on age, gender, and postmortem interval (PMI) to match the COVID cases and were collected by the MUSC brain bank. All individuals with DS COVID + cases in this study were treated in the intensive care unit for severe COVID and two of the three cases died from COVID-related complications. Case #24 was a 50-year-old male who had severe COVID, treated at the ICU, after which he recovered and was COVID negative (PCR test) and returned to an assisted living facility. During the subsequent year, his AD symptoms worsened, and the patient passed away one year following the COVID-19 infection. All DS cases with COVID were confirmed with a PCR test. Among the Swedish cases, all but one of the 8 COVID-19-positive cases were identified with nasopharyngeal PCR — either during hospital care or *postmortem* prior to autopsy. One COVID case was not tested but was later found positive—it was primarily sent for prion disease diagnostics due to suspected Creutzfeldt-Jakob’s disease. In this cohort symptoms varied extensively, likley due to various comorbidities. Six of eight cases were in-hospital patients, treated in the ICU or within the department of infectious disease, while one came to the emergency department as an unexpected cardiac arrest, and one died at home.

**Table 1:** Demographic information

Case	Diagnosis	Age	Gender	Braak	CERAD	COD	Comments
1	AD COVID-	90	F	VI	C	Acute pneumonia and heart failure	Late onset AD
2	AD COVID-	96	F	V-VI	C	Unknown	Late onset AD
3	AD COVID-	76	F	V-VI	C	Natural progression of dementia	Late onset AD
4	AD COVID +	67	M	VI	C	COVID-pneumonia and heart failure	Pneumonia/cardiovasc
5	AD COVID +	71	F	III	B	COVID-pneumonia aspiration and cardiac arrest	Vascular component in brain COVID-related vasoplegia and minor bleeding
6	AD COVID +	71	F	IV	B	Aortic rupture	Several cer. infarcts, older
7	AD COVID +	87	M	VI	C	Active COVID-19 infection with fever at the time of his demise	Diffuse LBD, CAA mild to moderate, atherosclerosis moderate to severe
8	CTRL COVID-	53	F	0	0	Cardiac pulmonary aneurysm	N/A
9	CTRL COVID-	74	M	0	0	Multi sys org fail	N/A
10	CTRL COVID-	59	M	0	0	Cardiac arrest	N/A
11	CTRL COVID-	30	M	0	0	Renal failure	N/A
12	CTRL COVID-	56	F	0	0	Respiratory failure	N/A
13	CTRL COVID +	37	M	0	0	Epileptic seizure–cardiac arrest	Cardiovascular and Epilepsy
14	CTRL COVID +	71	M	0	0	COVID-pneumonia and heart failure	Pneumonia and cardiovascular
15	CTRL COVID +	65	M	0	0	COVID-pneumonia – ARDS	Acute respiratory distress syndrome
16	CTRL COVID +	33	F	0	0	Sudden grand mal seizure	Edema w. acute necroses, in ICU 1 month before death
17	CTRL COVID +	56	M	0	0	Epileptic seizures /COVID encephalitis	Sudden confusion, in hospital > 2 months. Epilepsy treatment unsuccessful
18	DS COVID-	37	M	0	0	Failure to thrive post-surgery	Diffuse plaques, Thal phase 3
19	DS COVID-	64	F	0-II	A	Cardiac failure	N/A
20	DS-AD COVID-	66	M	V-VI	C	Dementia	N/A
21	DS-AD COVID-	58	M	V-VI	C	Dementia	CAA frequent
22	DS COVID +	31	M	0	0	DS with severe COVID, died from cardiac condition	Frequent diffuse plaques, mild CAA/ cardiovascular symptoms
23	DS COVID +	35	F	0	0	DS severe COVID/pneumonia	Mild hippocampal atrophy
24	DS COVID + recovered	50	M	VI	C	Dementia-related COD	DS with mild dementia before COVID, died 1 year after of severe dementia

The AD COVID + and Control-COVID + cases were collected at Lund University, the DS COVID + cases were collected at University of Colorado and the DS-AD, Control, and AD cases without COVID were collected at the MUSC brain bank in Charleston and the DSBC. These collaborating sites use harmonized brain bank collection protocols and adhere to the NIH-AA brain banking protocols [6, 40].

### Tissue preparation

Brain pathology assessment was performed on paraffin-embedded cortical and limbic tissue as well as the medulla and spinal cord using a harmonized procedure based upon the NIA-AA protocol [6] with a few modifications at each site (Lund University, MUSC, UC Irvine, and CU Anschutz). The cases from DSBC were obtained under existing IRBs at each site and using a standard operating procedure [3]. Brains were carefully removed, and a standard assessment was conducted of the gross anatomy including regional atrophy and inspection of major blood vessels for vascular disease to document potential comorbidities or previous central events of importance for diagnosis. Brains were photographed from multiple surfaces and sliced in 1 cm coronal slices using a plexiglass brain jig to produce uniform coronal slices at each site [3]. The slices were placed on a cutting board and photographed prior to microdissection for freezing (left hemisphere) and immersion fixation (right hemisphere) for microscopic analysis. Right hemisphere slices were fixed in 4% paraformaldehyde for 72 h and transferred to a cryoprotectant solution consisting of 30% sucrose in phosphate buffered saline (PBS) with sodium azide according to a harmonized protocol [3]. Data regarding time and date of death, date of birth, potential clinical diagnoses, *PMI*, wet weight of the entire brain, and any special *antemortem* circumstances such as anxiety or pain were recorded. All cases were assigned a randomly generated global unified identifier (GUID). A neuropathology report was conducted on 19 standard brain regions consistent with a modified NIA-AA protocol [3, 43]. Dissected fixed brain regions were paraffin embedded [65], cut at 5-microns and stained for hematoxylin and eosine (H&E), silver impregnation, and immunohistochemistry using alpha-synuclein, amyloid, phospho-tau, TDP43, and other specialty stains according to NIH protocols. For more details regarding the harmonized protocols in the DSBC consortium, see [3]. The cohort from Sweden underwent a clinical diagnostic analysis [10, 44] as performed in every-day diagnostic autopsies (700 investigated cases per year). Briefly, the AD cases included patients who had a clinical diagnosis of Alzheimer-related dementia in the clinic followed by a neuropathological diagnosis as outlined in the NIA-AA protocol, see e.g., [10]. One laboratory (Barrow Neurological Institute) also cut 40-micron thick sections from fixed cryoprotected slabs on a sliding microtome for immunohistochemistry [65].

### Immunostaining for the SARS-CoV-2 nucleoprotein

**Fig. 1:**
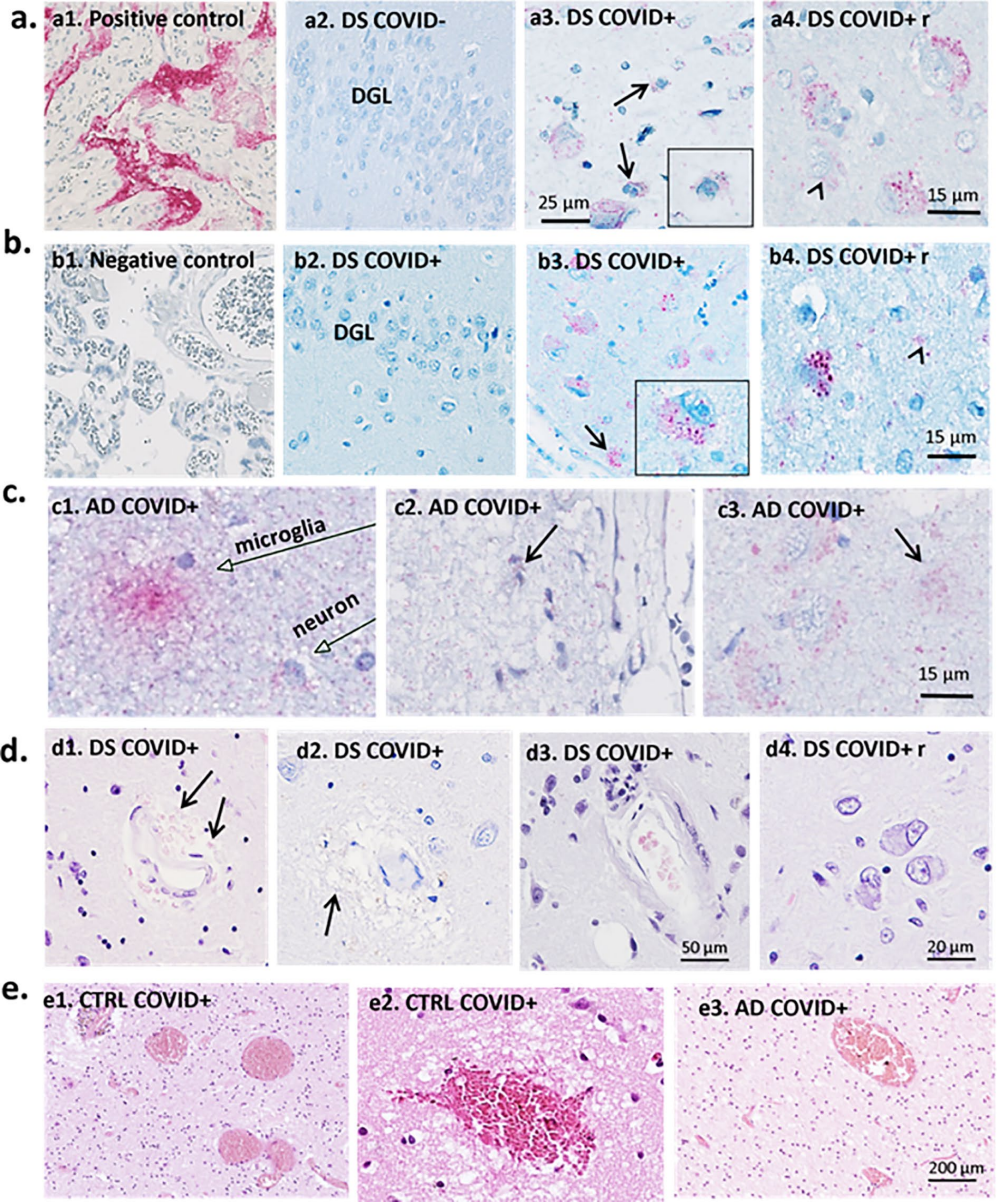
SARS-CoV-2 nucleoprotein immunostaining and H&E histochemistry. **a**–**c** SARS-CoV-2 nucleoprotein immunostaining (red stain) in positive (a1) and negative (b1) controls, AD and DS cases. a1 is a positive control (human placenta) and b1 is a negative control (human placenta). a2 is a micrograph from the dentate gyrus of a 37-year-old negative DS control (case #18 in Table 1). a3 and a4: SARS-CoV-2 immunoreactivity in neurons and glia cells (arrows in a3 and a4) in the frontal cortex in a 31-year-old DS COVID + case. The a3 inset shows a glial cell displaying punctate SARS-CoV-2 nucleoprotein cytoplasmic staining in a DS COVID + case. a4 displays SARS-Cov-2-positive neurons and microglia in layers II-III of frontal cortex from 50-year-old male with confirmed COVID infection a year before death but negative at death (DS COVID + *r*). a2–4 shows SARS-CoV-2 nucleoprotein immunostaining in the hippocampus of a 35-year-old who died from acute COVID (b2), the 31-year-old DS COVID + case (b3), and the 50-year-old who recovered before death (DS COVID + *r*, b4). Note the lack of nucleoprotein immunoreactivity in the dentate gyrus granule cell layer (DGL) of the hippocampus in the 35-year-old case (b2) compared to the strong positive staining observed in the 31-year-old acute DS COVID case (b3) and the recovered case (b4), suggesting a temporal spread of the virus from frontal cortex to the hippocampus. The inset in b3 shows strong punctate SARS-CoV-2 labeling in a CA1 neuron in the hippocampus. Arrows in b3 and b4 mark glial cells labeled with the nucleoprotein antibodies. c1–c3: SARS-CoV-2 nucleoprotein labeling in cortex of AD COVID + cases from autopsy at Lund University. Note a strongly positive microglial cell in c1, and arrows pointing to positive glial cells in c2 and c3. C2 depicts positive cells (red labeling) in Layer I of the frontal cortex. Since this layer is devoid of neurons, positive cells in this layer represent glial cells only. **d** and** e** H&E hippocampal staining showing vascular abnormalities including microbleeds (d1, arrows) and extravasation of erythrocytes (d2 and d3) and vacuolization (arrow d2) as well as inflammatory infiltrates (d3) and neurodegenerative changes including dying and pyknotic neuronal cell bodies (d4) in all three DS COVID + and the DS COVID + r (d4) cases. e1–e3: H&E staining shows vascular abnormalities including focal congestion-like vein dilatation (e1, e3) and mild rupture of the vein wall with limited perifocal bleeding (e2) in the frontal cortex of a control COVID + and an AD COVID + case, respectively. Sections shown in panels** a–c** were counterstained with hematoxylin. Scale bar in a3 represents 25 microns in a1-3; scale bar in a4 represents 15 microns; scale bar in b4 represents 15 microns for b1-4; scale bar in c3 represents 15 microns for c1–3; scale bar in d3 represents 50 microns for d1–3; scale bar in d4 represents 20 microns and scale bar in e3 represents 200 microns for e1-3

An anti-SARS-CoV-2 nucleoprotein antibody was used to detect the virus in *postmortem* brain tissue. The antibody (Catalog # 40,143-V08B; NP_828858.1; Met1-Ala422, Sino Biological (Nordic Biosite)/40143-T62) was produced in rabbit immunized with purified, recombinant SARS-CoV Nucleoprotein and used at a dilution of 1:2000. The staining was conducted using the Roche Ventana pathology laboratory staining system with Ultra View Universal AP (alkaline Phosphatase) Red Fast Red B as the chromophore. Positive/negative controls included staining of PCR-verified/excluded SARS-CoV-2 in paraffin-embedded fixed placental tissue (Fig. 1, a1 and b1, respectively). Sections were counterstained with Hematoxylin. The nucleoprotein antibodies were tested against an anti-double-stranded RNA antibody [J2] (ab288755, Abcam), and tested for cross-reactivity with herpes simplex virus (HSV) 1 and 2, and cytomegaloviruses (CMV). The staining for the SARS-CoV-2 virus was conducted for all the cases at Lund University. Confirmatory studies included immunostaining with an anti-spike protein SARS-CoV-2 antibody (Proteintech, Rosemont, IL).

### Neuropathologic and clinical diagnostic evaluation

Neuropathological staging was conducted according to the Alzheimer’s disease neuropathologic change (ADNC) protocol, considering Braak, CERAD, and Thal diagnostic scoring [3, 6, 9, 43]. For cases obtained from Lund University (AD + COVID and Control + COVID), the diagnosis of AD and of vascular pathology, as well as the exclusion of such pathology in the controls and other pathology, was established in adherence with internationally accepted criteria for AD [15, 40, 60, 76]. The diagnostic strategy was corroborated by the extensive clinical information available in these cases (see Table 1), as described in a previous publication [44].

### Immunohistochemical staining

Standard H & E and immunohistochemistry using primary antibodies directed against alpha-synuclein (cat: AB5038, Millipore, Darmstadt, Germany, 1:250), Aβ 1–42 (Abcam, cat. AB5078P, 1:100), and phospho-Tau (AT8; cat: MN1020, Thermo Fisher Scientific, Waltham, MA, USA, 1:250) was performed. Endogenous peroxidase activity was blocked by a 7:2:1 ratio of TBS, MeOH, and 3% hydrogen peroxide (H_2_O_2_), respectively, followed by incubation with 100 mM sodium metaperiodate. Pretreatment with heat and 0.05% citraconic anhydride for 10 min were used to enhance p-Tau and amyloid immunostaining. Sections were blocked with TBS containing 0.25% Triton-X and 10% normal serum for 1 h and incubated overnight with the primary antibody. After washing in PBS, sections were incubated with biotin-conjugated secondary antibodies, washed in PBS, incubated with streptavidin–horseradish peroxidase (HRP) complex, and visualized using a 1 mg/mL 3′,3′-diaminobenzidine (DAB) solution containing 0.02% H_2_O_2_, dehydrated, and cover-slipped using Permount mounting medium. Immunohistochemical controls included deleting either the primary or secondary antibodies resulting in no detectable reactivity. Immunohistochemical stains from the cases obtained from each brain bank were performed together in the same batch to avoid inter-batch variabilities in staining.

The tissue sections shown in Fig. 3 were obtained from additional 1 cm brain slices fixed in 4% paraformaldehyde for 48 h, cryoprotected in graded concentrations of sucrose, sectioned on a freezing sliding microtome at 40-micron thickness and immunostained at Barrow Neurological Institute. All other sections were generated from 5-micron paraffin-embedded blocks obtained on a microtome and were stained at CU Anschutz Medical Campus. This manuscript is the result of a collaboration between several different laboratories with slightly different protocols.

### Glial staining and analysis

Two to three sections per brain region were immunostained with antibodies directed against glial fibrillary acidic protein (GFAP, rabbit polyclonal, Abcam, Boston, MA, cat. # EPR1034Y, 1:500) to visualize astrocytes, a pan-microglial marker Iba-1 (rabbit polyclonal, WAKO, Richmond, VA, cat. # 013–27691, 1:1,000) and a transmembrane protein 119 (TMEM119) specifically expressed by resident microglia (rabbit polyclonal, Abcam, Waltham, Boston, MA, cat. # ab185333, 1:100), but not macrophages [5], following the above immunohistochemistry protocol. Iba-1 and TMEM119 immunostained sections were in addition counterstained with hematoxylin. All sections included in the quantification were stained at CU Anschutz Medical Campus and parallel staining with the same antibodies were also conducted at Barrow Neurological Institute.

Three images of each region of interest (ROI) were captured on each section using a Nikon Optiphot microscope for image analysis at 20X magnification. The ROIs examined included gray matter of the middle frontal gyrus (Brodmann area 46), hippocampal dentate gyrus and CA1 subfield, and the para-hippocampal gyrus. For each ROI, three images of the underlying white matter were captured for comparison with gray matter. Images used for quantification were taken at the same magnification and light intensity.

### Sholl analysis

Since the complex morphologic structure of astrocytes is crucial to their situational function in brain, we determined group differences in peri-vascular astrocytic processes and branching using the Fiji ImageJ software package with the Sholl analysis plugin [25, 71]. This assessment was performed by an unbiased investigator blinded to the groups. Sholl is an open-source software used for morphometric analysis of astrocytic processes by quantifying length, number, and intersections at concentric spheres originating from the soma [74]. The first 25 astrocytes in the vicinity of a blood vessel in gray matter displaying a visible nucleus and at least one process stained for GFAP were analyzed per section and per region by an investigator blinded to the identity of each sample. Morphologic complexity of the astrocytes was calculated based on the number of intersections at each radial distance from the starting point, including sum of intersections, mean intersections, and ramification index determined by the Sholl plugin software [25, 71]. The ramification index is the ratio between the maximum number of intersections of the processes with the circles and the number of the primary processes.

Iba-1 immunostaining was evaluated by an unbiased and blinded investigator using the Fiji ImageJ software system (Version 2.3.0/1.53q) according to a semiquantitative previously validated and published protocol [14]. First, the color channels were split to isolate the DAB-reaction product from background staining. The threshold was adjusted to include only immunostained cells, and % area stained was assessed. Measurements were taken on three random areas per section of the dentate gyrus, CA1, and the white matter adjacent to the hippocampal formation.

### Statistical analysis

Brain tissue from each case was stained 2–4 different times, allowing multiple sections to be evaluated per brain and ROI. In addition, repeated staining batches ensured that all groups were represented in each staining batch to avoid inter-batch variability in staining density between groups. A nested two-way ANOVA was used, where one factor (individual sections in this example) was nested within another factor (infection/diagnosis) due to the limited access to COVID-19-infected brains from each group in the study. We used a nested design to determine the morphologic complexity of single astrocytes [1]. Nested designs yield clusters of observations that are not considered independent and require special considerations in terms of statistical analysis [1]. Valid and efficient subgroup analyses were performed using nested case–control data according to previously published protocols [20]. This nested case–control design provides an unbiased estimate of the effects of SARS-CoV-2 neuroinflammation in several samples of each brain region, using several different parameters obtained from the Sholl analysis of astrocytes, and the density of two different microglial markers. Previous work has shown that subgroup analyses provide unbiased estimates of the effects in a population, when sampling different brain regions and different distinct neuronal populations from the same individual as carried out in the current study [20]. Sex, age, and *PMI* were considered in the analyses, but none altered the statistical outcomes.

### Ethical considerations

*Postmortem* consents were obtained from the next of kin for all brain donations used in the study, either via the DSBC consortium or the Carroll Campbell Jr. Neuropathology Laboratory (CCNL) brain bank at the Medical University of South Carolina (MUSC). For the Swedish cohort, ethical permission was given from the regional ethical authorities, nr 2020–02369. The application was submitted since the intention of the primary examination (autopsy) was a clinical diagnostic investigation.

## Results

### SARS-CoV-2 nucleoprotein and H&E staining

Positive (Fig. 1a1) and negative (Fig. 1b1) immunostaining for the SARS-CoV-2 nucleoprotein showed significant staining in the placenta from a PCR-confirmed patient (Fig. 1a1) and a PCR-confirmed negative placenta (Fig. 1b1). SARS-CoV-2 nucleoprotein immunoreactivity appeared granular within glial cells (Fig. 1a3) and neurons (Fig. 1a3, a4,) in frontal cortex of all three DS COVID-19 + cases. In the hippocampus, two of the three DS COVID + cases, the 50-year-old (Fig. 1b4) and the 31-year-old (Fig. 1b3) were positive for SARS-CoV-2, but not the 35 YO case with DS and COVID + (Fig. 1b2). SARS-CoV-2 nucleoprotein immunoreactivity was not observed in the frontal cortex (not shown) or hippocampus in individuals with DS without (Fig. 1a2) COVID-19 infection. Interestingly, the strongest nucleoprotein immunostaining was seen in both the frontal cortex (Fig. 1a4) and hippocampus (Fig. 1b4) of the DS case that died 12 months after contracting COVID-19 but tested negative for the virus at the time of death (DS COVID + r). SARS-CoV-2 nucleoprotein immunolabeling was also observed in all AD and control COVID-19 + cases (Fig. 1c1-3). A microglial cell in gray matter is shown with strong nucleoprotein immunostaining in an AD COVID + case in Fig. 1c1. The sections were counterstained with hematoxylin (blue). Pink granular labeling represents SARS-CoV-2 nucleoprotein immunostaining.

### Brain histopathology

Vascular abnormalities including microbleeds and extravasation of erythrocytes (Fig. 1d1-2) as well as inflammatory infiltrates and deposits or calcification of the external wall of the blood vessels (Fig. 1d3) were observed in the hippocampus and frontal cortex in all three DS COVID + patients. This was confirmed by the neuropathology report for the cases, where the two DS cases with acute COVID-19 were found to have mild CAA and microcalcifications, respectively (see Table 1). Abnormal neuronal morphology with pyknotic cell bodies (Fig. d4) was frequently observed in DS COVID + cases. Focal blood vessel abnormalities were often seen in both gray and white matter in the AD COVID + and Control-COVID + cases, with scattered aggregates of congestion-like dilated veins (Fig. 1e1 and e3), rupture of the vein wall (Fig. 1e2) and limited perifocal bleeding (Fig. 1e2). Some vascular abnormalities were associated with tissue edema (Fig. 1e2). Such vascular alterations were not seen in the non-COVID cases.

### p-Tau and amyloid immunostaining

**Fig. 2:**
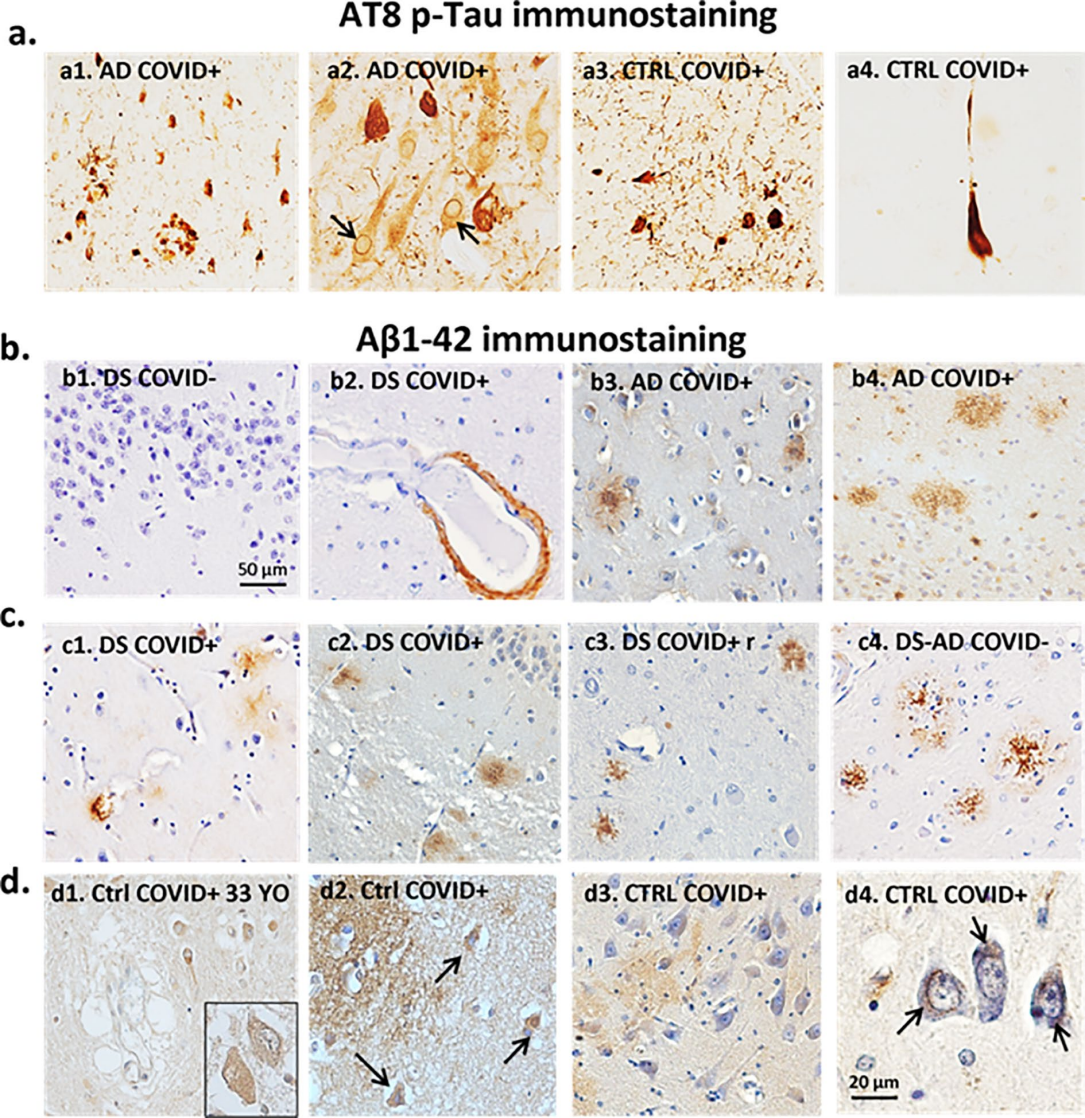
AT8 and Aβ1-42 immunostaining. **a** Images showing AT8 positive NFTs in the frontal cortex (a1) and hippocampus (a2-a4). a1-a2. AD COVID + cases contained frequent NFTs (dark brown staining) and lightly labeled neurons with an intact morphology (i.e., pre-tangles) in frontal cortex and hippocampus (arrows), respectively, compared to a few scattered NFTs in control COVID + cases (a3, a4). Note numerous AT8 positive neuropil threads in the hippocampus of a 71-year-old control COVID + case (Fig. 2a3) compared to a rare NFT observed in the CA1 of a 65-year-old patient with COVID (Fig. 2a4). **b** and** c** Aβ1-42 (brown) immunostaining in AD, DS, and control cases. b1. Aβ immunostaining was not observed in DG of the hippocampus of a young person with DS (37-year-old) without COVID-19. b2–b4. Images showing an occasional Aβ positive blood vessel in the frontal cortex (b2) of a DS COVID + case, and numerous fibrillar and diffuse amyloid plaques in the AD COVID + cases (b3 and 4). **c**. Frontal cortex diffuse Aβ (c1-c2) in DS COVID + cases compared to neuritic plaques in the DS COVID + r case (c3) and a DS-AD COVID- case (c4). The DS-AD case depicted in c4 was a 66-year-old male with DS, while the DS COVID + cases in c1 and c2 were 31 and 35 years old, respectively, and the DS COVID + r case in c3 was 50 years old. **d** Weak to moderate deposits of diffuse (d2, age 37 and d3, age 71 years) and intracellular (d1, age 33, d2 age 37 (arrows) and d4, age 65 (arrows) Aβ1-42 immunostaining were seen in the CA1 and dentate gyrus of the hippocampus in 5 out of 5 control COVID + cases (d1-4). All sections in panels b-d were counterstained with Hematoxylin. The scale bar in b1 represents 50 microns for a1, a3, b1, b3, c1-c4, and scale bar in b4 = 20 microns in b4, a2, and a4

All AD and DS cases with a Braak stage of V-VI exhibited p-Tau (AT8) NFT-like immunostaining in the frontal cortex (Fig. 2a1), hippocampus (Fig. 2a2), and para-hippocampal gyrus (not shown). In the two AD cases with Braak stage III and IV all-tau pathology was observed in limbic structures. In addition, several (Fig. 2a3) to occasional (Fig. 2a4) AT8 positive neurons and neuropil threads were found in the para-hippocampal gyrus but not in the hippocampal formation or frontal cortex in two control COVID + cases (71 and a 65-years-old, see Table 1).

Aβ-positive plaque pathology was not observed in the dentate gyrus (DG) of the hippocampus of a young person with DS (37-year-old) without COVID-19 (Fig. 2b1), which had a Thal Phase score of 3 with mild cortical atrophy (Table 1). An occasional Aβ positive blood vessel in the frontal cortex (Fig. 2b2) as well as diffuse amyloid plaques (Fig. Fig. 2c1-2) were observed in the 31 YO and the 35 YO cases with DS and COVID-19. On the contrary, frequent fibrillar and diffuse amyloid plaques were observed in the 50 YO male who passed away with severe dementia one year after COVID-19 (DS COVID + r, Fig. 2c3). The frequency of amyloid plaques in this 50 YO case was comparable to that seen in DS-AD cases without COVID infections (Fig. 2c4). Finally, we found diffuse amyloid plaques in the neuropil (Fig. 2d2 and 3) and intra-neuronal (d4, arrows and Fig. 2d1, inset) Aβ labeling in the hippocampus of control COVID + cases, respectively. Intraneuronal and extracellular Aβ immunostaining was observed in all young (Case #13, 16 and 17) and the two older (Case# 14,15) Control-COVID + cases (see Table 1).

### GFAP astrocytic immunostaining

**Fig. 3:**
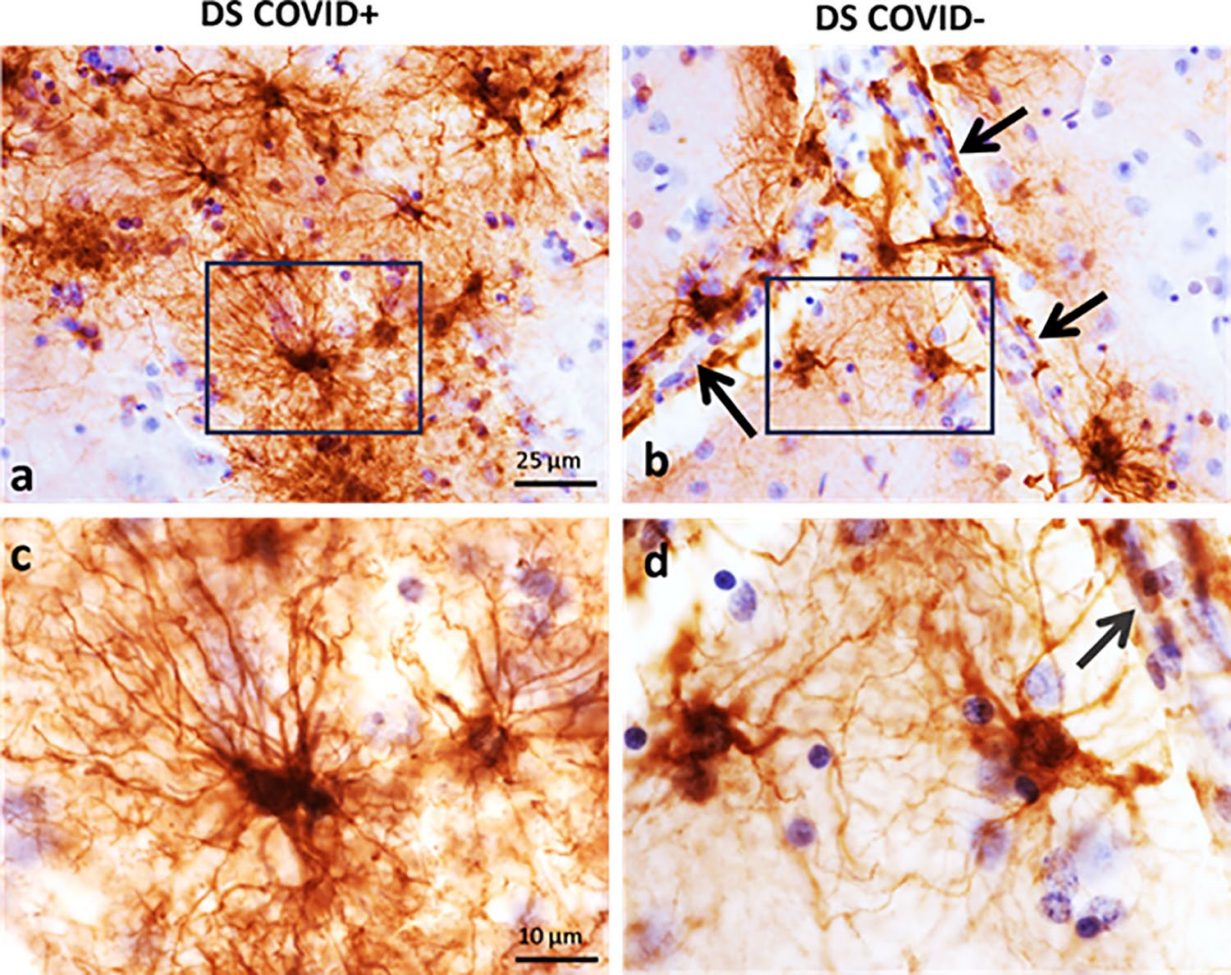
GFAP immunostaining in the frontal cortex. Low (**a** and **b**) and high (**c** and **d**) magnification photomicrographs showing GFAP positive astrocytes in the frontal cortex gray matter of a 31-year-old DS COVID + (**a**, **c**) compared to a 37-year-old DS COVID- (**b**, **d**) case. Panels **c** and** d** show a higher magnification image of the boxed area in **a** and** b** displaying an increase in the length and number of GFAP positive processes in a DS COVID + (**C**) compared to a COVID- (**d**) DS case. Black arrows in **b** and **d** indicate the close apposition of GFAP processes with the vascular wall. All sections were counterstained with H&E. The scale bar in **a** = 25 µm applies to **b**, and bar in **c** = 10 µm, which applies to **d**

**Fig. 4:**
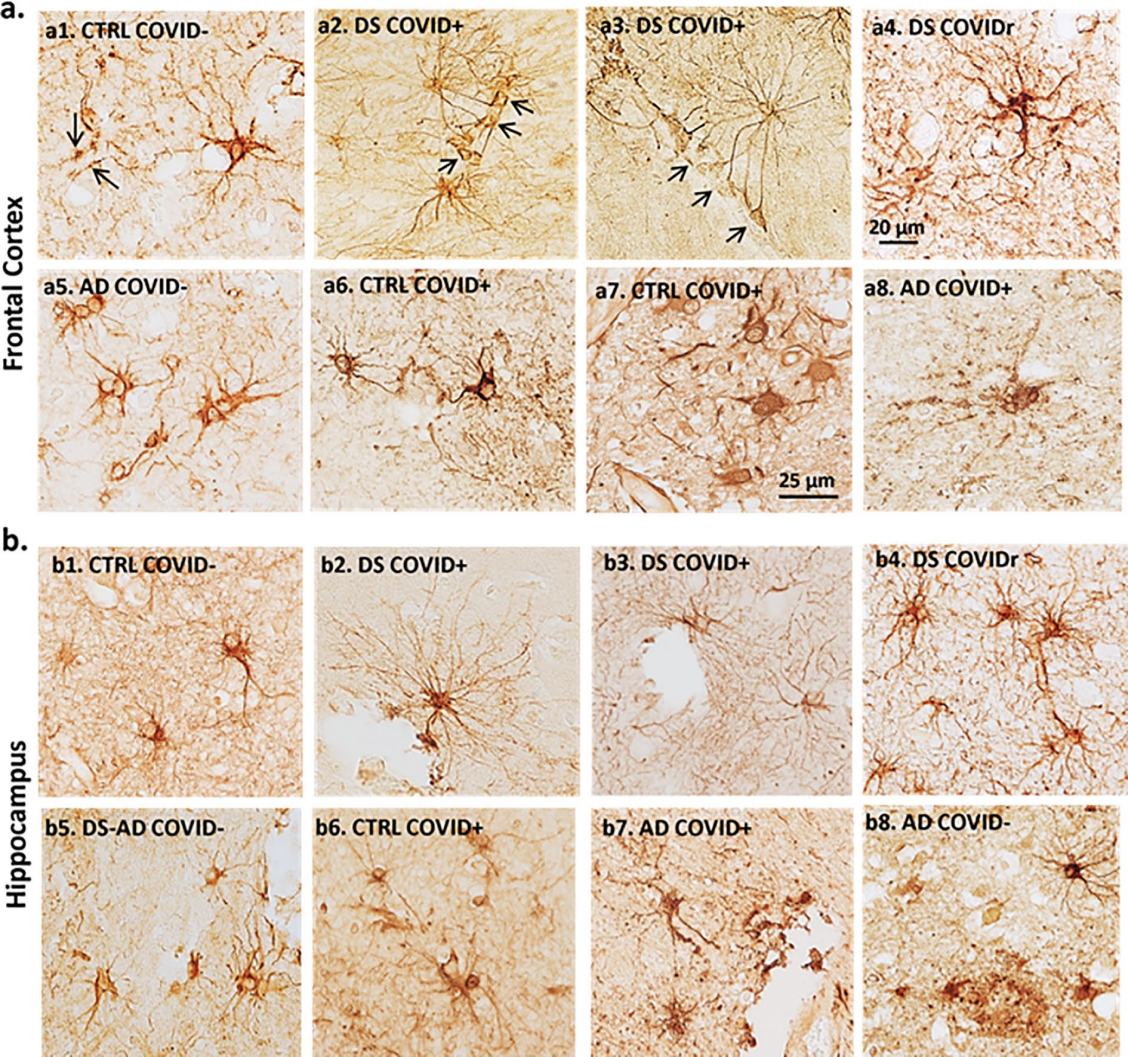
GFAP immunostaining in the frontal cortex (**a**) and hippocampus (**b**) across cases. Frontal cortex (a1-8) and hippocampal (b1-8) images showing differences in morphology between GFAP labeled peri-vascular astrocytes (brown) in DS COVID + compared to other cases. GFAP positive glia exhibited long processes with prominent end-feet in close apposition to the vascular wall (arrows) in DS COVID + cases (a2,3 and b2,3) compared to smaller cell bodies and fewer slender branched processes in the frontal cortex (a1, arrows) and hippocampus (b1) in control non-COVID cases. AD (a5, b8) and DS-AD (b5) COVID- cases displayed GFAP positive astrocytes with thicker but shorter processes. Control COVID + (a6, a7, b6) and AD COVID + (a8, b7) cases exhibit reduced GFAP astrocytic activation and occasionally displayed an apoptotic or necrotic cell (a8, b7). The scale bar in a5 represents 20 microns for all panels except for a8, where the scale bar represents 10 microns

GFAP immunostaining revealed different astrocytic morphologies between cases with and without COVID-19 (Figs. 3 and 4). Representative images of GFAP immunostaining in the frontal cortex from a 31-year-old male with DS who died from severe COVID-19 (Fig. 3a and c), compared to the frontal cortex from an individual with DS without dementia and no COVID-19 infection (Fig. 3b and d). Note the extensive dense branching of individual astrocytic processes in COVID + (Fig. 3a and c) compared to shorter and less dense GFAP stained processes surrounding the bifurcation of a blood vessel in a DS COVID-(Fig. 3b and d) case.

Similar astrocytic morphologies were observed in frontal cortex (Fig. 4a2-4) and hippocampus (Fig. 4b2-4) in DS COVID-19 + cases. Control non-COVID-19 cases (Control COVID-) showed star-shaped peri-vascular astrocytes with small end-feet extending onto the vascular wall in the frontal cortex and hippocampus (Fig. 4a1 and b1). AD and DS-AD COVID-19 negative (COVID-) cases exhibited an increase in the density of GFAP labeled cells, with shorter, stubbier processes indicative of an inflammatory glial-induced response to the AD pathology (Fig. 4a5, b5, b8). By contrast, AD COVID + and DS COVID + cases displayed elongated astrocytic processes and a significant increase in the number of processes extending from each cell within the gray matter of the frontal cortex (Fig. 4a2-4,a5) and hippocampus (Fig. 4b2-4, b7). This was particularly evident adjacent to the vascular wall in the gray matter in both regions. Moreover, peri-vascular astrocytes also extended enlarged end-feet, particularly in the DS COVID + cases, sometimes surrounding the vascular wall (Fig. 4a2 and a3, arrows). Glial processes extended long distances reaching the vascular wall, where prominent end-feet lined the lumen, resembling a protective wall of end-feet known as *glia limitans*. This pattern of astrocyte processes and prominent end-feet on the vascular wall resembles that seen in encephalitis [69], but not usually observed in AD or DS brain tissue. In some areas of gray matter, GFAP staining appeared punctate, and an occasional degenerating cell body was seen mainly in the AD COVID + cases (Fig. 4a8 and b7). The DS recovered from COVID-19 case (Fig. 4a4 and b4) displayed astrocytic morphologies like that seen in the hippocampus and frontal cortex of AD COVID- (Fig. 4a5) and DS-AD COVID- (Fig. 4b5) cases, suggesting that acute SARS-CoV-2 infection gives rise to the abnormal astrocyte morphology observed in DS COVID + and AD COVID + cases, which does not linger months after recovery from acute infection.

**Fig. 5:**
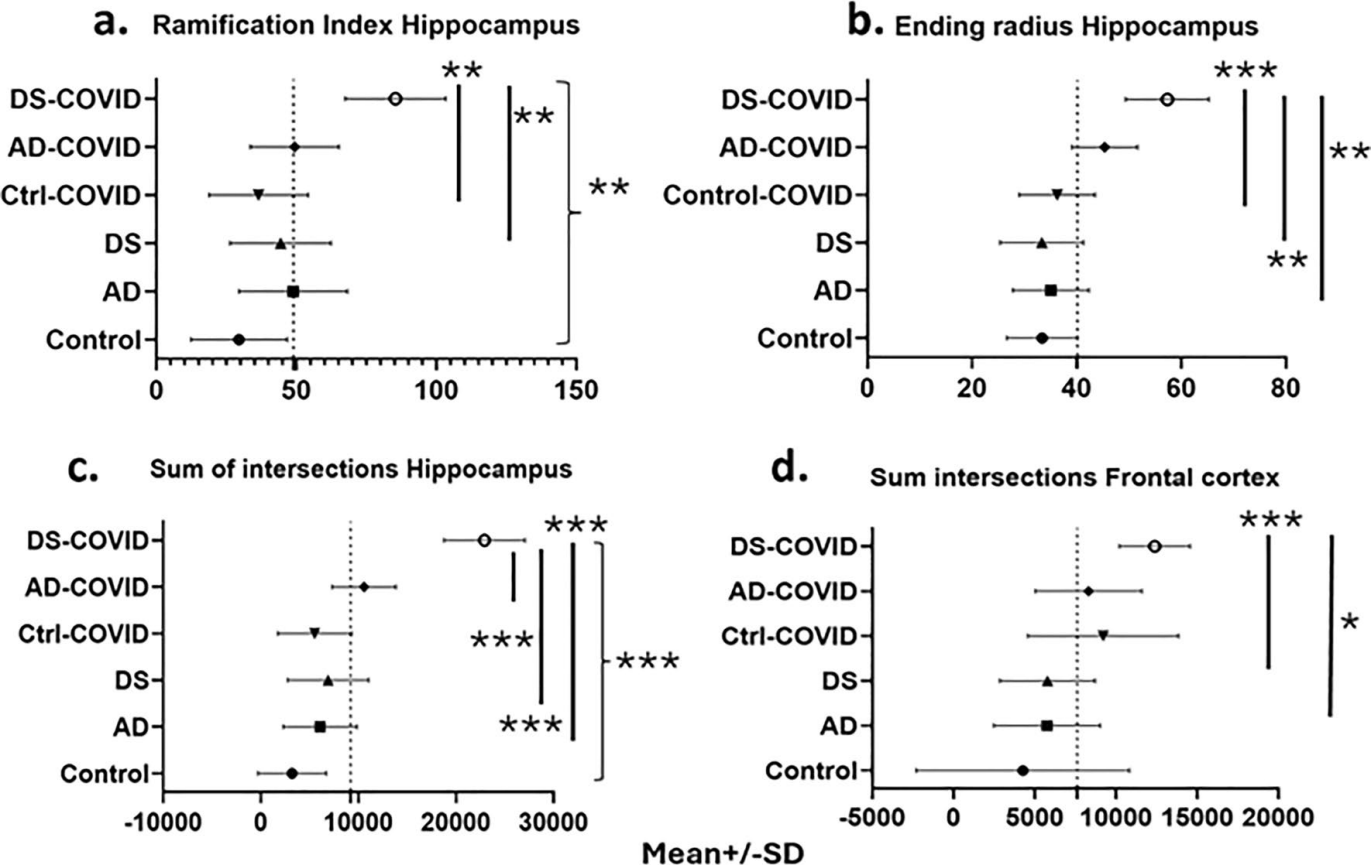
Sholl analysis of differences in astrocytic morphology across groups. **a** Analysis revealed a significantly higher ramification index for peri-vascular astrocytes in the gray matter in DS COVID + group compared to DS COVID- (Tukey test, *p* = 0.04, *n* = 24) and control non-COVID (Tukey test, *p* = 0.004) and COVID + (Tukey test, *p* = 0.01) groups. **b** Ending radius measurements of peri-vascular astrocytic processes were significantly greater in DS COVID + compared to control COVID + (Tukey test, *p* = 0.0007), AD COVID- (Tukey test, *p* = 0.002), and DS COVID- (Tukey test, *p* = 0.001) groups. **c**. Analysis of the sum of intersections in the hippocampal gray matter revealed significant differences between groups (Nested ANOVA, *p* =  < 0.0001) with greater numbers of astrocytic intersections in the DS COVID + group. The Tukey post-hoc test revealed significant differences between control COVID- vs. DS COVID + (*p* < 0.0001), AD COVID- vs. DS COVID + (*p* < 0.0001), DS COVID- vs. DS COVID + (*p* < 0.0001), control COVID + vs. DS COVID + (*p* < 0.0001), and AD COVID + vs. DS COVID + (*p* = 0.0006). **d** Nested ANOVA analysis revealed significant differences in the sum of intersections only for frontal cortex gray matter between groups; Tukey post hoc test revealed a greater sum of intersections in DS COVID + compared AD COVID-, *p* = 0.02) and DS COVID- (*p* = 0.01) groups

Sholl analysis of GFAP immunostained sections revealed an increase in arbor complexity in peri-vascular astrocytes in DS and AD COVID-19 positive cases. The ramification index for peri-vascular astrocytes in the gray matter of hippocampus is shown in Fig. 5a. The ramification index is the ratio between the maximum number of the intersections of the astrocytic processes and the number of primary astrocytic processes [25]. The average hippocampal ramification index in the control COVID- was 29.6 ± 5.6, the AD COVID- was 44.2 ± 15.2, the DS COVID- was 41.1 ± 16.7, the control COVID + was 36.7 ± 4.2, the AD COVID + was 47.1 ± 18.3, and the DS COVID + was 84.2 ± 0.8. The highest average ramification index was observed in the DS- COVID + compared to the other groups, with nearly a threefold higher average in the control COVID- cases. The overall ramification index in the hippocampus between groups had a p value of 0.007 (nested one-way ANOVA F, dfd 5.745, 5, 11, where F is the F distribution, dfn is the degrees of freedom of the numerator, and dfd is the degrees of freedom of the denominator). A post-hoc analysis (Tukey’s method) revealed a significantly higher average ramification index in DS COVID + compared to non-COVID control (*p* = 0.004) cases, and between the DS COVID + vs. DS COVID- (*p* = 0.04) group. Finally, DS COVID + cases showed a significantly higher ramification index compared to the control COVID + group (*p* = 0.01). Although sex, age, and *PMI* were considered as covariates, statistical outcomes were not changed. Whether access to a larger group of DS COVID cases would alter the present statistical outcomes remains to be determined.

We also measured the ending radius of astrocytic processes. The DS COVID + exhibited the longest extension of astrocytic peri-vascular processes (mean length of 58 ± 7.8 μm) compared to the control COVID- (mean length of 35 ± 6 μm) group. AD COVID + group also exhibited greater astrocytic process length (mean of 46 ± 10 μm). Statistical analysis revealed an overall significant difference between groups (Fig. 5b, *p* = 0.0003; F, DFn, Dfd 6.509, 5, 31). Post-hoc group comparisons using the Tukey’s multiple comparisons test revealed significant differences between control COVID + and DS COVID + (*p* = 0.0007), AD COVID- and DS COVID + (*p* = 0.002), and DS COVID- and the DS COVID + (*p* = 0.001) groups, suggesting that the COVID-19 infection induced a significant lengthening of peri-vascular astrocytic processes, particularly in DS compared to control COVID- and AD COVID- cases.

A key measure of astrocytic complexity is the sum number of branch points of astrocytes. The average number of intersections was highest in the DS COVID + cohort, with an average sum of intersections of 23,125 ± 1391 compared to an average 3042 ± 1000 intersections in the control COVID- group. Sholl analysis of the sum intersections of GFAP positive astrocytes within the hippocampus revealed a significant difference between groups (Fig. 5c *p* =  < 0.0001; F, DFn, Dfd: 13.18, 5, 31). Post-hoc analysis revealed a statistical significance between control COVID- vs. DS COVID + (*p* < 0.0001), AD COVID- vs. DS COVID + (*p* < 0.0001), DS COVID- vs. DS COVID + (*p* < 0.0001), control COVID + vs. DS COVID + (*p* < 0.0001), and AD COVID + vs. the DS COVID + (*p* = 0.0006) groups.

We next measured morphologic alterations to astrocytes in the gray matter of the frontal cortex using Sholl analysis (Fig. 5d). Although differences in astrocyte morphology were observed between the groups in the frontal cortex (see Figs.3 and 4a), Sholl analysis revealed only a statistically significant change in the sum of intersections (Fig. 5d) (*p* = 0.007; F, DFn, Dfd 4.484, 5, 19). The average values for sum of intersections in the frontal cortex was 4270 for the control COVID- group, 5760 ± 871.6 for the AD COVID-, 5499 ± 2023 for the DS COVID, 9214 ± 2640 for the control COVID + , 8313 ± 1875 for the AD COVID + and 12,129 ± 1296 for the DS COVID + group. Post-hoc Tukey analysis revealed significantly higher numbers of intersections in the DS COVID + compared to the AD COVID- (*p* = 0.02) and DS COVID- (*p* = 0.01) groups in the frontal cortex. This analysis revealed the highest number of astrocyte branch points in DS COVID + , followed by control COVID + and then AD COVID + cases in this brain region (Fig. 5d), with more than a 100% increase in the average number of branch points in the DS COVID + compared to the non-COVID control group. Interestingly, both the control COVID + and the AD COVID + groups showed elevated average branch points compared to the control COVID- cases, suggesting a common feature of astrocytic morphology after SARS-CoV-2 infection.

### Iba-1 and TMEM119 microglial immunostaining

**Fig. 6:**
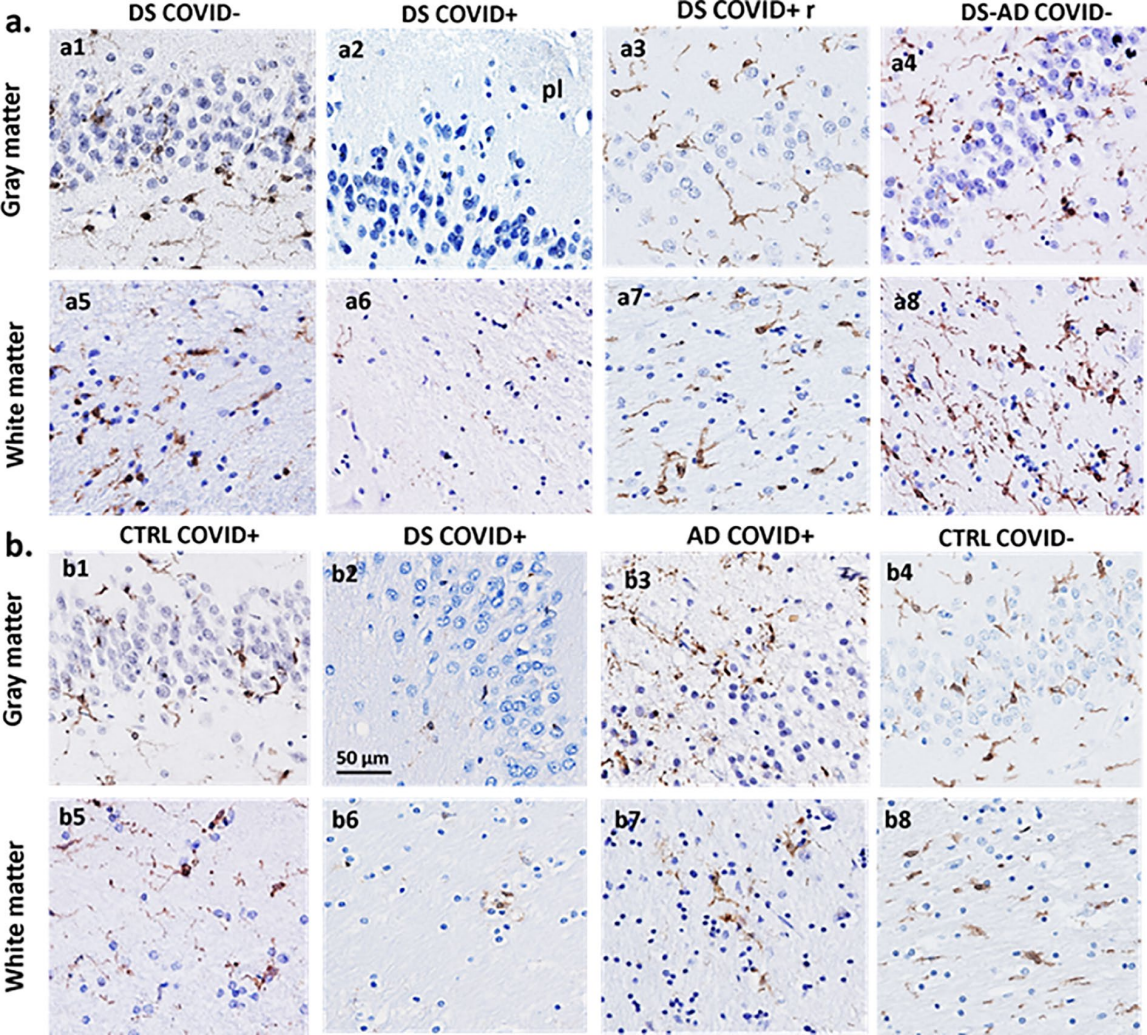
Hippocampal Iba-1 immunostaining in the dentate granule cell layer (DGL) and white matter between COVID + and COVID- cases. **a**. Images showing a reduction in Iba-1-ir cells in DGL (a1-a4) and white matter (a5-a8) in DS COVID + (a2, a6) compared to DS COVID- (a1, a5) and DS-AD COVID- (a4, a8) cases, while intermediate Iba-1 cell immunostaining was seen in the DS COVID + r (a3, a7) case. **b**. Images showing reduced Iba-1 positive cells in the DGL (b1-b4) and white matter (b5-b8) in a DS COVID + (b2) compared to control COVID + (b1), AD COVID + (b3) and control COVID- (b4) case. The DS COVID + case depicted in a2 and a6 was 31 years old, and the DS COVID + case depicted in B2 and B6 was 35 years old, respectively, while the DS COVID- case in A1 and A5 was 37 years old, and the DS COVID + r case depicted in a3 and a7 was 50 years old. The DS COVID- case shown in a1 and a5 was 37-years old. All sections were counterstained with Hematoxylin. Scale bar in b2 = 50 µm applies to all panels

We examined the morphology of microglial cells in the hippocampus and frontal cortex using antibodies against Iba-1 that recognize microglia and peripheral immune cells (Figs. 6 and 9) and TMEM119 (Figs. 8 and 9), which only label microglia [26]. Qualitative observations revealed fewer Iba-1 immunostained cells in gray and white matter in both frontal cortex and hippocampus (Fig. 6a2, a6, b2, b6) in DS COVID + cases compared to the other groups. The loss of Iba-1-ir microglia in the gray matter of DS COVID + (Fig. 6a2) seen in both the 31-year-old and the 35-year-old DS COVID + cases was conspicuous compared to the DS 37-year-old age-matched individual without COVID infection (Fig. 6a1). A similar loss of Iba-1 immunostaining was observed in the white matter (Fig. 6a6 and b6), but not to the same extent as observed in gray matter. Most groups showed a comparable density of Iba-1 positive microglia in white matter, except for an increase in the white matter of the DS-AD COVID- case (Fig. 6a8) compared to the other groups. Higher magnification microscopy revealed focal groups of activated Iba-1-ir microglia in the DS COVID + (Fig. 9b) group and numerous small, rounded Iba-1-ir cell bodies in the gray matter (Fig. 9b inset and c), compared to the more common branched microglial morphology observed in control COVID- brains (Fig. 9a).

**Fig. 7:**
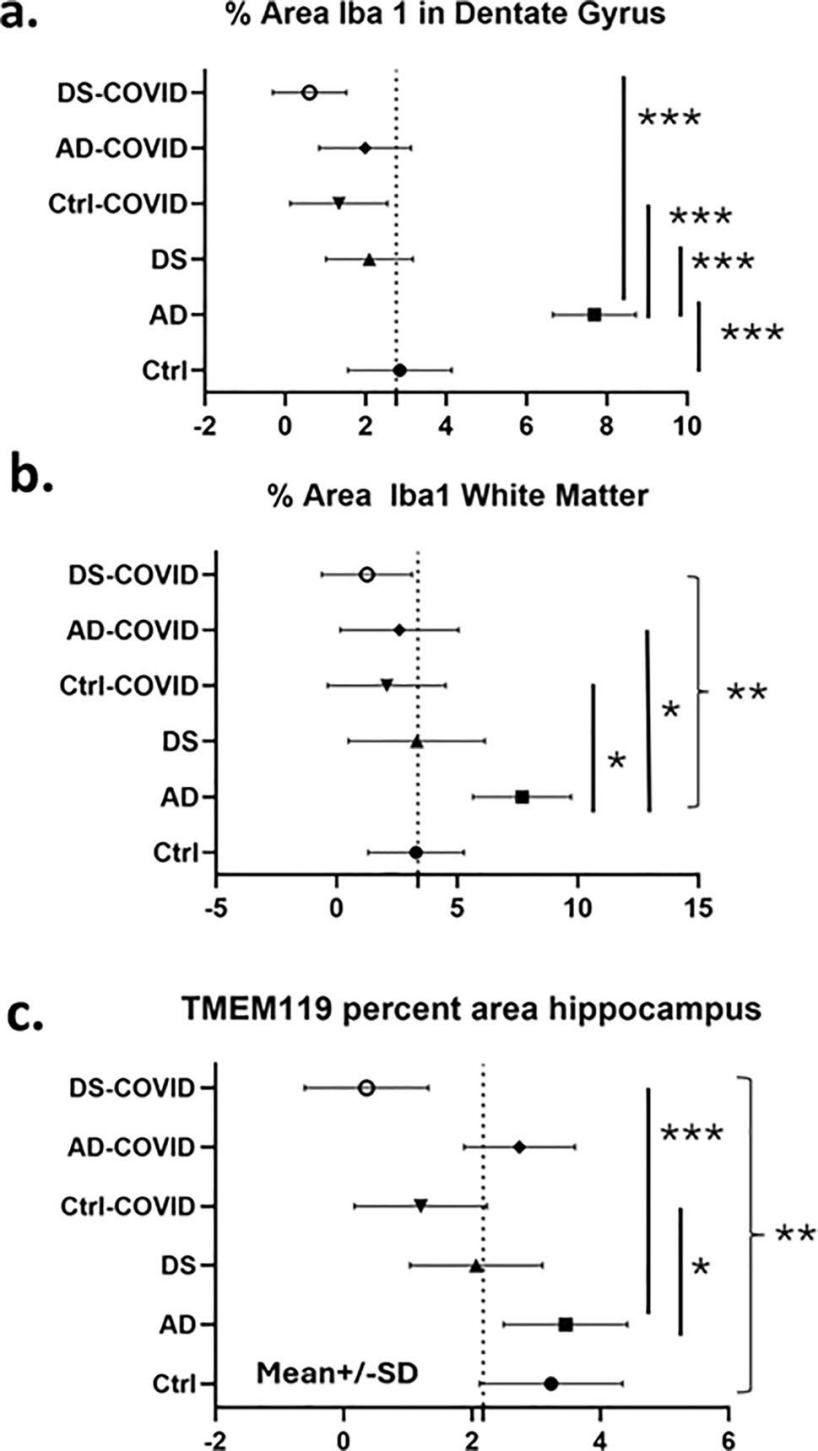
Analysis of Iba-1 and TMEM119 immunostaining density in hippocampal dentate gyrus and white matter. **a** Percent area of Iba-1 immunostaining was significantly lower in DS COVID + , DS COVID-, control COVID-, and control COVID + in hippocampal gray matter compared to AD COVID- cases (Tukey test, *p* < 0.0001 for all groups in comparison to the AD COVID- group, *n* = 20). **b** Measurements of the percent area of Iba-1 staining in the white matter was significantly greater in AD COVID- compared to DS COVID-, control COVID + , AD COVID + , DS COVID + groups (Tukey–Kramer test, *p* < 0.0001 for all groups). **c** Percent immunostaining in hippocampal white matter as examined using a nested ANOVA analysis. Nested one-way ANOVA, *p* value 0.0003 *p* value summary***, *F*, DFd, Dfd 6.2, 5, 40. Significance: DS COVID vs. AD COVID *p* = 0.0076, DS COVID vs. AD *p* = 0.0006, DS COVID vs. Ctrl, *p* = 0.0041, Ctrl-COVID vs. AD: *p* = 0.0279

To quantify the loss of Iba-1 and TMEM119 immunoreactivity in the hippocampus, we used a nested ANOVA analysis, where one factor (individual sections in this example) was nested within another factor (infection/diagnosis), to explore whether the percent area covered by Iba-1 positive microglia differed in the gray matter (Fig. 7a) or in white matter (Fig. 7b) between groups. The average percent area covered by Iba-1 positive microglia in the hippocampal gray matter was 2.5 ± 0.8 percent in control COVID-, 6.3 ± 4 percent in AD COVID-, 2.1 ± 0.1 percent in DS COVID-, 0.9 ± 0.9 percent in control COVID + , 1.9 ± 1.2 percent in AD COVID + , and 0.6 ± 0.3 percent in DS COVID + cases. We found a statistically significant difference between groups in hippocampal gray matter (nested ANOVA (*p* < 0.0001; F, DFn, Dfd = 24.60, 5, 53). Post-hoc analysis Tukey test showed significant group differences between the control COVID- and AD COVID- (*p* < 0.0001), AD COVID- and DS COVID- (*p* < 0.001), AD COVID- and control COVID + (*p* < 0.0001), and AD COVID- compared to DS COVID + (*p* < 0.0001) groups. These findings suggest that the COVID-19 infection affected Iba-1 labeled immune cells in the gray matter in both the AD and the DS cases, with the greatest loss in the DS COVID + cases, which displayed an approximately fourfold lower percentage of staining compared to the control COVID- group. Whether similar statistical differences between microglia densities would be found in larger cohorts of similar patient groups remains to be seen.

In the hippocampal white matter (Fig. 7b), the overall percent area covered by Iba-1 positive cells was also significantly different between the groups following a nested ANOVA analysis where individual sections are nested within groups (infection vs. diagnosis; *p* = 0.005, F, DFn, Dfd = 5.627, 5, 14). Post-hoc Tukey test revealed significant differences between the AD COVID- vs. control COVID + (*p* = 0.02), AD COVID- vs. AD COVID + (*p* = 0.04) and AD COVID- vs. DS COVID + (*p* = 0.002) groups. The average percent area covered by Iba-1 positive cells in the white matter was 3.9 ± 1.7 percent in control COVID-, 5.98 ± 5 percent in AD COVID-, 3.3 ± 1.1 percent in DS COVID-, 1.8 ± 0.9 percent in control COVID + , 2.75 ± 0.4 percent in AD COVID + , and 1.2 ± 0.8 percent in DS COVID + cases.

**Fig. 8:**
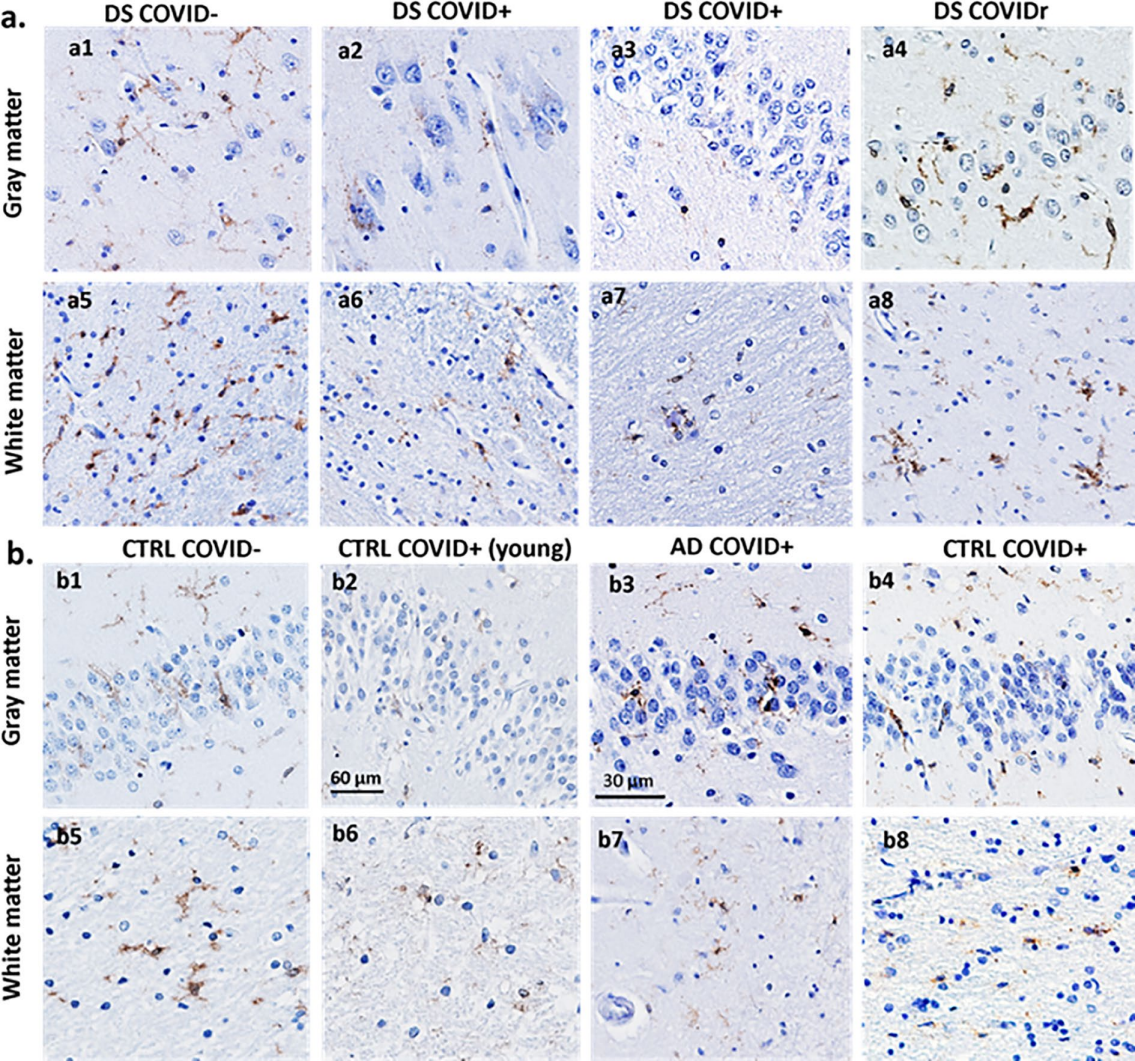
Microglial TMEM119 immunoreactivity in gray and white matter of the hippocampus. **a** Images showing a reduction in TMEM119-ir cells in the CA1 region (a2) and the DGL (a3) and white matter (a6, a7) in DS COVID + (a2, a3, a6, a7) compared to a DS COVID- (a1, a5) and DS COVID + *r* (a4, a8) case. **b** Images showing less TMEM119 immunoreactivity in the DGL (b2) and white matter (b6) of the hippocampus in a young control COVID + (33-year-old) compared to control COVID- (59-year-old) (b1, b5), AD COVID + (71-year-old) (b3, b7) and an older control COVID + (71-year-old) (b4, b8) case. All sections were counterstained with Hematoxylin. The scale bar in b3 represent 30 microns in a2, a3, a4, and b3, and the scale bar in b2 represents 60 microns for the rest of the panels

**Fig. 9:**
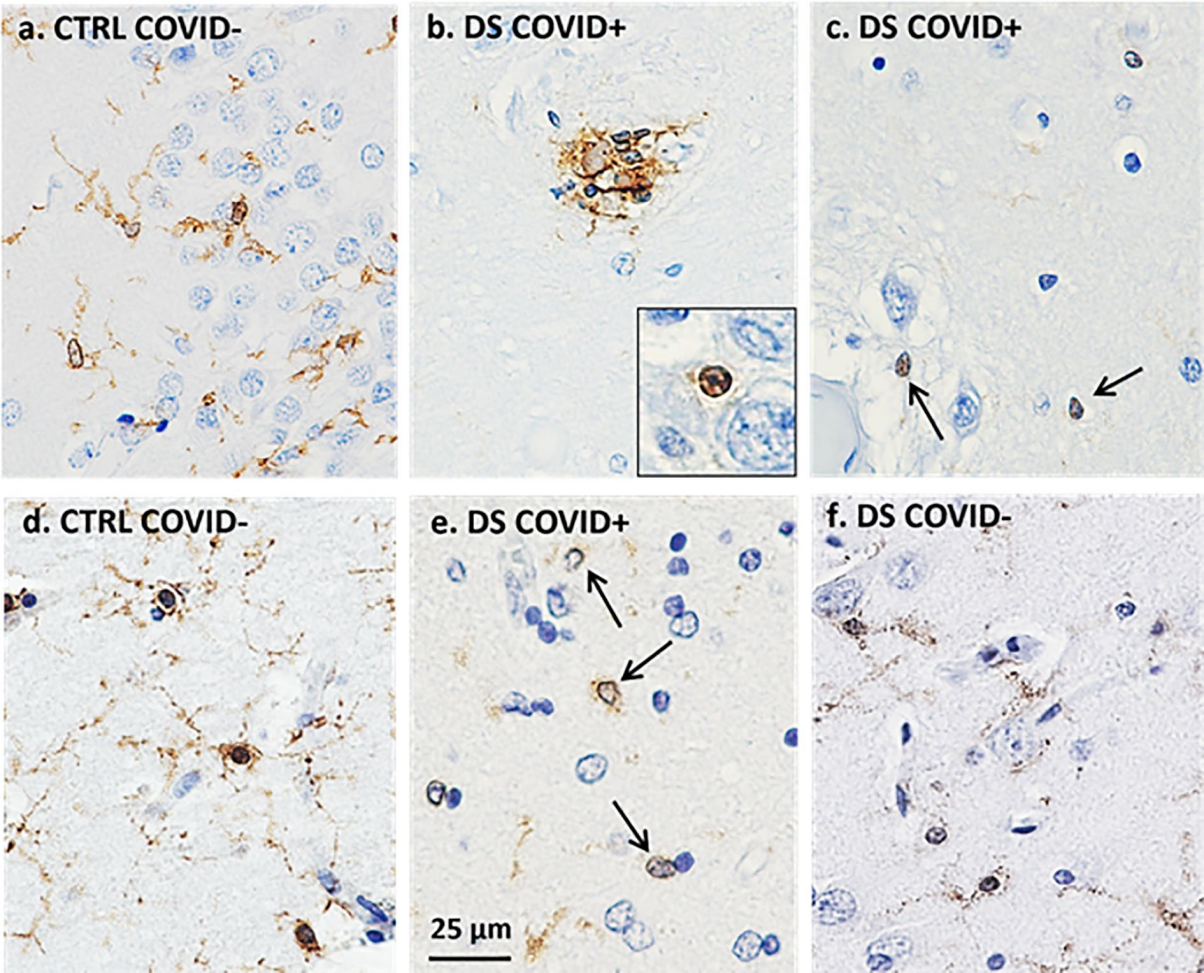
Morphologic features of Iba-1 (**a–c**) and TMEM119 (**d**–**f**) positive hippocampal microglia in control COVID- and DS COVID + cases. Microglial cells stained for Iba-1(**b**, **c**) and TMEM119 (**e**) displayed a rounded appearance with few processes (arrows in **c** and **e,** and inset in **b**), resembling the morphology observed in phagocytic microglial cells, in DS COVID + (**b**, **c**, **e**) compared to more elongated cell bodies and extensive processes seen in control COVID- (**a**, **d**) and DS COVID- (**f**) cases. Clusters of Iba-1 immunostained microglial cells were occasionally observed in white matter (**b**) in DS COVID + cases. All sections were counterstained with Hematoxylin. The scale bar in d represents 25 microns for all images

There was also a reduction in the density of TMEM119 stained microglia in the CA1 pyramidal cell layer (Fig. 8a2) and DG (Fig. 8a3) of the hippocampus in the DS COVID + and control COVID + (Fig. 8b2) groups. A nested ANOVA revealed an overall significant difference between the groups (*p* = 0.0003; F, DFn, and Dfd were 6.2, 5 and 40, respectively, see Fig. 7c). However, no significant difference between the groups was found in terms of percent staining for TMEM119 in white matter of the same cases. A greater number of TMEM119-positive cells were found in the white matter than in the gray matter in all groups (Fig. 8). TMEM119-positive microglia displayed rounded, smaller cell bodies with few observable processes in DS COVID + (Fig. 9e) or a resting-type morphology with longer, slender processes, mostly in control COVID- or in DS COVID- (Fig. 9d and f, respectively) cases. In the hippocampal gray matter of the DS COVID + cases, TMEM119-positive rounded cell types were more prevalent than those with a resting-type morphology (Fig. 9e), suggesting activation of remaining microglia in response to the SARS-CoV-2 infection. Focal groups of activated microglia were observed in both gray and white matter in the DS COVID + (Fig. 9b), and sometimes in AD COVID + and control COVID + cases (data not shown).

## Discussion

This is the first systematic study comparing the neuropathological effects of SARS-CoV-2 infections in *postmortem* brain tissue obtained from patients with DS, AD, and non-demented healthy controls compared to non-COVID counterparts. SARS-CoV-2 nucleoprotein immunostaining, using a nucleoprotein antibody against the virus [75] demonstrated viral nucleoprotein in neurons and microglial cells in gray matter of the frontal cortex and hippocampus of two of the three DS cases with confirmed SARS-CoV-2 infection. GFAP immunostained astrocytes exhibited a significant increase in length of arbors, as well as a significant increase in the numbers of branch points on astrocytic processes in DS COVID + and AD COVID + compared to the other groups, particularly in peri-vascular astrocytes. These findings suggest that SARS-CoV-2 infection gives rise to a significant alteration in peri-vascular astrocyte morphology, consisting of an overall lengthening of processes and a significant increase in branch points in individuals with DS COVID + and AD-COVID + post infections, as well as an increase in the size and number of vascular end-feet. In addition, there was a reduction of Iba-1 and TMEM119 stained microglial cells in gray matter but to a lesser extent in white matter in the frontal cortex and hippocampus.

SARS-CoV-2 nucleoprotein displayed a granular appearance within neurons and glial cells in the frontal cortex and hippocampus in SARS-CoV-2 infected DS cases. Recently, it was shown that both neuron-derived and astrocyte-derived exosomes obtained from patients with COVID-19 contain both SARS-CoV-2 Spike protein 1 and nucleocapsid (N) proteins, suggesting that exosomes play a role in transport and/or cellular uptake of the virus [64]. A recent study demonstrated that exosomes from SARS-CoV-2 infected lungs reach the brain parenchyma, which affects AD associated neuronal gene regulatory networks in frontal cortex, temporal cortex and hippocampus in AD accelerating neurodegeneration implicating an importance of brain exosomes in disease progression [2]. To confirm exosomal content of viral particles future studies will undertake sub-cellular co-labeling of the nucleoprotein with vesicle markers in DS and AD. We found SARS-CoV-2 weak to moderate nucleoprotein immunostaining in the frontal cortex of all three DS cases with COVID-19, but only in the hippocampus in two of the three DS cases—the case that had the infection 12 months prior to death, and the patient that passed away acutely from severe COVID-19. However, whether this coronavirus enters the central and/or peripheral nervous system to directly affect blood vessels, neurons, and glial cells (see [27]) or are secondary to the cytokine storm [22] remains controversial. In this regard, it has been shown that the BBB is dysregulated in COVID-19 + which serves as a potential entry route for SARS-CoV-2 to the brain [51]. These investigators also demonstrated SARS-CoV-2 in the basolateral compartment of the BBB using a trans-well assay after apical infection in vitro, suggesting active replication and transcellular transport of the virus across the BBB perhaps by exosomes [2], or via other systems.

In a recent study, 41 autopsy cases with confirmed COVID-19 showed areas from each brain with hypoxic/ischemic changes, which were either global or focal with large and small infarcts, many of which were hemorrhagic [75]. The presence of viral RNA and protein, using quantitative reverse-transcriptase PCR, and immunocytochemistry with primers, probes and antibodies directed against the spike and nucleoprotein regions, found low but detectable viral RNA levels in brain tissue but were unable to detect immunohistochemical evidence of viral particles [75]. On the other hand, another autopsy study provided evidence for peri-vascular hemosiderin-laden macrophages and hypoxic-ischemic changes in neurons [52], which we also observed (data not shown). Although immunostaining for SARS-CoV-2 viral spike and nucleoprotein was seen in a single brain, PCR revealed SARS-CoV-2 RNA in all brains [52]. In another autopsy study, confocal imaging of sections stained for fluorescence, RNAscope, and immunohistochemistry demonstrated extracellular SARS-CoV-2 virions but failed to show viral particles in the brain parenchyma or olfactory bulb [50]. However, whether the patients examined had AD and/or DS was not reported. This is important because individuals with DS and/or AD have a deficient BBB and an altered brain-immune system [53, 58]. These deficiencies likely would affect the invasiveness of a virus or other pathogens representing a potential factor for the increased mortality to COVID-19 found in these two patient groups. Other autopsy studies have shown similar findings as those presented here in the AD brain post COVID-19 infection. For example, Reiken and collaborators found inflammatory activation in the brain of COVID-19 patients, as well as activation of pathways related to tau phosphorylation—indicating that AD pathology may be accelerated by the viral infection [67]. In addition, Poloni and colleagues showed that patients with a pre-existing neurocognitive syndrome suffered from delirium after contracting COVID-19, which was related to hyperactivation of microglia in the brainstem and hippocampus [66]. These novel findings together with the present observations support the need to explore DS and/or AD-related COVID-19 pathology across a wide range of clinical–pathologic cases.

A recent study provides additional information regarding the neuro-invasiveness of the SARS-CoV-2 virus and its variants [17]. Investigators infected golden hamsters with the original Wuhan SARS-CoV-2 strain, and with the Gamma, Delta and Omicron/BA.1 viral strains and demonstrated that all viral variants were neuro-invasive and were retrogradely or anterogradely transported along axons in the brain [17]. de Melo et al. [17] showed neuro-invasiveness of all three viral variants studied to date. It is important to consider whether differences in staining protocols, fixation, or PMI affects staining for the virus, its spike protein or nucleoprotein antibodies in brain.

The findings reported here indicate that astrocytes around the brain vasculature display an intense immune response, particularly in those with DS that died from a SARS-Cov-2 infection or severe COVID-19-related complications. Astrocytic end-feet surrounding blood vessels play an important role in viral neuropathogenesis [70]. This was especially evident in the DS brains with COVID-19, where peri-vascular astrocytes presented with significantly enhanced vascular end-feet, which enveloped the entire lining of the vascular wall of a single cell (Fig. 4, arrows). Astrocytes are thought to represent a viral reservoir in the brain [47], which is supported by images showing infection in the brain of the long-term DS COVID survivor (Fig. 1**)**. The current data suggest an increased immune response in the brain of those with DS and COVID-19, which may explain the more severe complications observed in the clinic in individuals with DS. Interestingly, microglia express ACE2 receptors [45] which may make them especially vulnerable to a SARS-CoV-2 viral attack. Although direct infection of SARS-CoV-2 virus into microglial cells in vitro induces a microglial inflammatory response followed by cell death [45], conclusive evidence of this occurring in *postmortem* brain in humans remains to be demonstrated but is partially supported by the findings reported in the current study.

Many of the COVID-19 brains investigated here showed either a greater or reduced accumulation of hemosiderin-laden macrophages in areas of vascular injury in the frontal cortex and hippocampus. To differentiate macrophage from microglia cells, we used an Iba-1 antibody that recognizes both macrophages and microglia and an antibody against TMEM119 that is specific to brain-derived microglial cells [26, 68]. We found that both markers were reduced in the gray matter in the frontal cortex and hippocampus in DS COVID + cases, whereas others reported increases in the number of nodes of activated microglia labeled with Iba-1 and alterations in BBB integrity in *postmortem* brains of patients with AD and COVID-19 [29]. Although these previous findings suggest that COVID-19 infection in AD increases the number of microglia in the brain, this report did not differentiate between gray and white matter, nor did it include the distribution of GFAP positive astrocytes. Here, we observed similar focal accumulations of Iba-1 positive microglial cells, particularly in the white matter underlying the hippocampus (see Fig. 9b). Although anecdotal reports suggest that neurodegenerative disease is accelerated by COVID-19 disease, the consequences of SARS-CoV-2 infections were not systematically examined in the brain of people with DS, adding to the novelty of the present report.

Although neuro-invasion of SARS-CoV-2 viral particles into brain is still debated, human autopsy and animal model studies indicate that the virus has significant effects on brain function, leading to neurologic symptoms and long-term disabilities at all ages [27]. Autopsy studies have revealed significant cardiovascular involvement, including large vessel strokes, hemorrhagic microbleeds, extravasation and BBB disruption, and peripheral T-cell and macrophage invasion in the parenchyma [82] caused either by direct viral attack of glial cells and neurons or indirectly via a massive cytokine storm resulting from acute infection [62]. The current study provides novel information regarding effects of this virus in the brain of people with DS without dementia compared to patients with AD or controls that succumbed to COVID-19.

This initial investigation has some limitations. The present study contains a small number of cases. The DS group consisted of three cases with a history of COVID-19, which were younger (50, 31, and 35 years old) compared to the age when AD pathology normally occurs in individuals with DS. Another limitation concerns the lack of a greater number of age-matched DS controls. Moreover, the relatively low number of COVID + controls (*n* = 5) hinder the ability to exclude the potential effects of other clinical covariates (e.g., hypoxia or seizures) (see Table 1) upon the interpretation of the present findings. The low number of cases is partially due to the almost complete cessation of autopsies for research purposes during the pandemic, which hopefully will be remedied by the continued expansion of the DSBC.

## Conclusions

The current preliminary observations presented here demonstrate a long-term viral presence as well as significant glial pathology in the brain of cases with DS and COVID-19 but to a lesser extent in patients with AD or controls with COVID-19. Although neuro-invasion of SARS-CoV-2 viral particles into brain is still debated, human autopsy and animal model studies indicate that the virus has significant effects on brain function, leading to neurologic symptoms and long-term disabilities at all ages. The current findings provide neuropathological data related to the long-term effects of the SARS-CoV-2 virus on brain inflammatory responses and AD-related pathology, which may play a role in cases with long-COVID and its neurologic symptoms. The present findings need to be expanded upon and confirmed in a larger controlled cohort of cases.

## Data Availability

The materials will be available to other research groups after publication. Brain tissues from the cases utilized in the current study can be available via the Down Syndrome Biobank Consortium, DSBC.
